# The structural and magnetic properties of dual phase cobalt ferrite

**DOI:** 10.1038/s41598-017-02784-z

**Published:** 2017-05-31

**Authors:** Shyam K. Gore, Santosh S. Jadhav, Vijaykumar V. Jadhav, S. M. Patange, Mu. Naushad, Rajaram S. Mane, Kwang Ho Kim

**Affiliations:** 1 Center for Nanomaterial & Energy Devices, School of Physical Sciences, Swami Ramanand Teerth Marathwada University, Nanded, 431606 India; 2 Dnyanopasak Shikshan Mandal’s Arts, Commerce and Science College, Jintur, 431509 India; 3 Materials Research Laboratory, Srikrishna Mahavidyalaya, Gunjoti, Omerga, Osmanabad, 413613 (MS) India; 4 Department of Chemistry, College of Science, Bld#5, King Saud University, Riyadh, Saudi Arabia; 5 Global Frontier R&D Center for Hybrid Interface Materials, Pusan National University, San 30 Jangjeon-dong, Geumjeong-gu, Busan 609-735 Republic of Korea

## Abstract

The bismuth (Bi^3+^)-doped cobalt ferrite nanostructures with dual phase, i.e. cubic spinel with space group Fd3m and perovskite with space group R3c, have been successfully engineered *via* self-ignited sol-gel combustion route. To obtain information about the phase analysis and structural parameters, like lattice constant, Rietveld refinement process is applied. The replacement of divalent Co^2+^ by trivalent Bi^3+^ cations have been confirmed from energy dispersive analysis of the ferrite samples. The micro-structural evolution of cobalt ferrite powders at room temperature under various Bi^3+^ doping levels have been identified from the digital photoimages recorded using scanning electron microscopy. The hyperfine interactions, like isomer shift, quadrupole splitting and magnetic hyperfine fields, and cation distribution are confirmed from the Mossbauer spectra. Saturation magnetization is increased with Bi^3+^-addition up to x = 0.15 and then is decreased when x = 0.2. The coercivity is increased from 1457 to 2277 G with increasing Bi^3+^-doping level. The saturation magnetization, coercivity and remanent ratio for x = 0.15 sample is found to be the highest, indicating the potential of Bi^3+^-doping in enhancing the magnetic properties of cobalt ferrite.

## Introduction

Structural and chemical composition of multi-component inorganic nanostructures have stimulated technological and scientific interest to alter the physiochemical properties while developing magnetic, electric, catalysis, and spintronic devices^1–6^. Spinel is one of the complex structures whose physical, magnetic and electrical properties can be altered by adding dopants and using the suitable route for the synthesis^7, 8^. The spinel ferrites have attracted considerable interest due to their use in microwave technology, magnetic storage, and biomedical applications etc.^3, 9, 10^. Spinel ferrite has general formula (A^II+^)[B_2_^III+^]O_4_^II−^, where A^II+^ and B^III+^ are the divalent and trivalent cations occupying tetrahedral (A) and octahedral [B] sites. Face-centered cubic structure of the ferrite is a result of cations and oxygen anions formulation. Divalent cation occupies either tetrahedral or octahedral sites, when it occupies tetrahedral sites, normal spinel is formed. On the other hand, when divalent cation occupies both tetrahedral as well as octahedral sites, inverse spinal is formed^11^. Similarly, a mixed structure can also be formed when divalent cation is distributed in both sites. The magnetic and electrical properties of ferrites depend on cation distribution and can be altered by varying the place of cation in the interstices. Cobalt and nickel ferrites (CoFe_2_O_4_ and NiFe_2_O_4_) are intensively studied spinel ferrites due to their high application potential^12, 13^. CoFe_2_O_4_ is the most versatile hard ferrite with mixed cubic spinel structure having Fd3m space group. CoFe_2_O_4_ exhibits high coercivity (5400 Oe), high magneto-crystalline anisotropy and moderate saturation magnetization^14–16^. Amongst several multiferroics, bismuth ferrite (BiFeO_3_) has been reported as one of the versatile cubic perovskites exhibiting both ferroelectricity and G-type anti-ferromagnetism above room temperature^17, 18^. In multiferroics, parameters such as electric polarization and magnetism generally are responsible for magnetoelectric effect^19^. The anti-ferromagnetism comes from unpaired electrons in the *d* shell of the Fe^3+^ with very weak ferromagnetic ordering due to canted spin structure. The ferroelectricity arises from the displacement of Fe^3+^ and Bi^3+^ in the unit cell^17^. These two effects are very weak in single phase material^19, 20^. Single phase multifunctional materials are rarely found in nature. Due to special structural features dual phase materials demonstrate different magnetic and electric properties. They demonstrate a strong multiferroic property and consequently have better application potential than single phase^21^. Thereby, dual phase materials have attracted much interest in research and industrial market. Such materials can be obtained by synthesizing artificial composite of ferromagnetic and ferroelectric materials^22^. Secondly, magneto-electric or multiferroic are important materials in recent years, where oxygen stoichiometry plays a crucial role in composition of oxides. The nonstoichiometry in oxide determines the phase stability and structural, magnetic and electrical properties of oxide materials^23^. These oxide materials have considerable demand in spintronic and data storage devices^24, 25^. In past, various investigators have reported the synthesis of either pure ferroelectric or ferromagnetic properties. To practical potential, it is necessary to develop dual phase composite materials. Synthesis of these materials is associated with the doping level and the choice of dopant.

We reported replacement of trivalent Fe^3+^ by Bi^3+^ in our previous work^26^. In the present paper, we report on the synthesis of dual phase Bi^3+^-doped CoFe_2_O_4_ nanostructures with general formula Co_1−x_Bi_x_Fe_2_O_4_ (CBF) where x = 0.0–0.2 by sol-gel self-combustion method. The change in the phase from cubic spinel to spinel-perovskite with the substitution of the Bi^3+^ in place of the Co^2+^ has thoroughly been investigated. Structural, morphological and magnetic properties of CBF as a function of Bi^3+^-doping levels are measured and reported. Presence of trivalent Bi^3+^ instead of divalent Co^2+^ in the CoFe_2_O_4_ crystal produces CBF with different stoichiometries, structures, morphologies and magnetic properties.

## Experimental Section

### Synthesis

The synthesis of CBF nanostructures was carried out by sol-gel self-combustion method with a range of x = 0.0 to 0.2. The high purity analytical reagent grade (99.99%) cobalt nitrate (Co(NO_3_)_2_ 6H_2_O), bismuth nitrate (Bi(NO_3_)_3_ 5H_2_O), ferric nitrate (Fe(NO_3_)_3_ 9H_2_O) and citric acid (C_6_H_8_O_7_ H_2_O) (sd-fine, India) chemicals were used as starting materials. All reagents were weighted in molar proportions; the products of the system were synthesized by keeping constant 1:3 metal nitrate to citrate ratio. The ferric nitrate, cobalt nitrate and citric acid were initially dissolved in de-ionized water and bismuth nitrate was dissolved in concentrated HCL to get clear and agglomeration-free solution. An aqueous solution of citric acid was mixed with metal nitrate as chelating agent^27^ and the pH of solution was increased to ∼7 by an addition of ammonia solution^28^. The solution was kept on hot-plate with continuous stirring at 90 °C. Due to evaporation process, the solution was turned to viscous and finally, a viscous gel was obtained. On removal of complete water molecules from the mixture, the gel was automatically ignited and burnt with glowing flints. The decomposition reaction would not stop until the whole citrate complex was consumed. The auto-ignition was complete within a minute, yielding the ashes termed as precursor product with some impurities, collected as sediment which was at the bottom of conical flask. The as-prepared powders (nanostructures) of all samples were heated separately at 500 °C for 5 h and further characterized.

### Characterizations

For the investigation of formation of the dual phase CBF composites, X-ray powder diffraction (XRD) patterns were recorded on Rigaku–denki (Japan) X-ray diffractrometer (D/MAX2500) using Cu-Kα radiation (λ = 1.5418 Å) in the 2*θ* range from 15 to 80° with scanning rate 10°/min. For examining the cross-out morphologies of samples and elemental composition percentage, involved in CBF, scanning electron microscope (SEM) digital photoimages and energy dispersive X-ray spectra (EDS) were used. The magnetic data for these samples were obtained with a vibrating sample magnetometer (VSM) at room temperature by Lake Shore: Model: 7404. The Mossbauer spectra were taken in transmission geometry at room temperature for which a ^57^Co/Rh γ-ray source was used. The velocity scale was calibrated relative to ^57^Fe in Rh. For the qualitative evaluation of the Mossbauer spectra recoil spectra were analyzed using WinNormosFIT software^29^.

## Results and Discussion

### Structural verification

**Figure 1:**
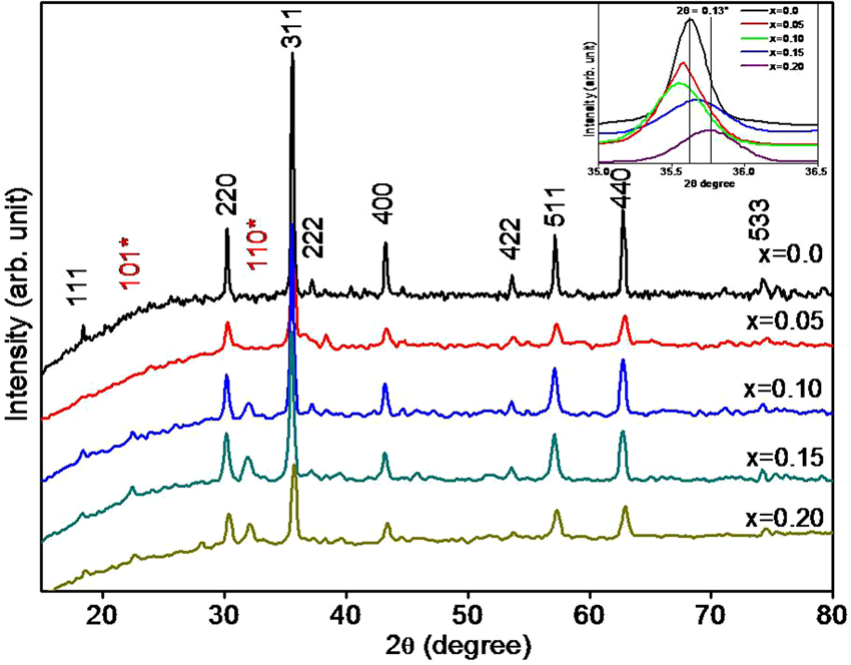
Powder XRD patterns of CBF for x = 0.0, 0.05, 0.10, 0.15 and 0.20. Inset is the (311) peak positions of CoFe_2_O_4_ for various Bi^3+^-doping levels i.e. various x values.

**Figure 2:**
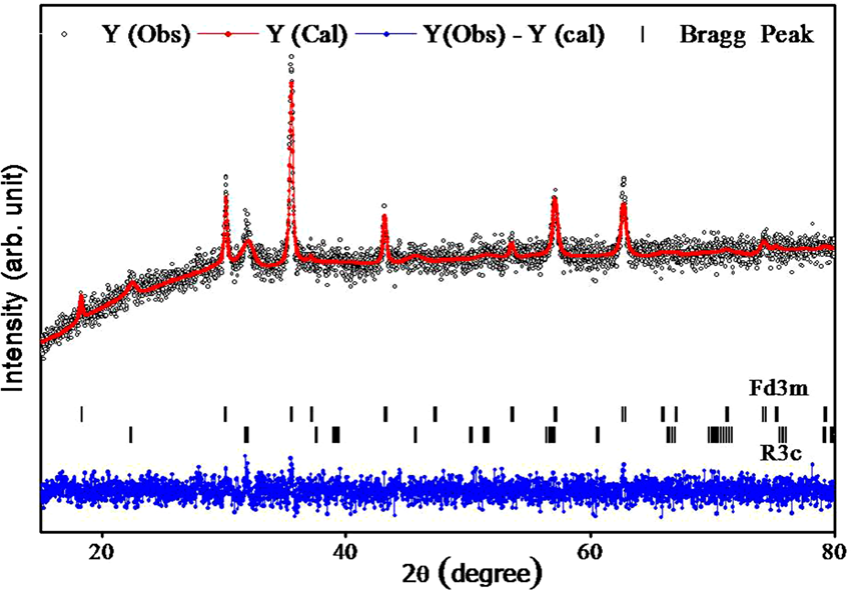
Rietveld refinement of CBF for x = 0.2 samples (dotted black lines are from Fd3m space group spinel phase and blue lines are due to R3c space group of perovskite phase).

**Figure 3:**
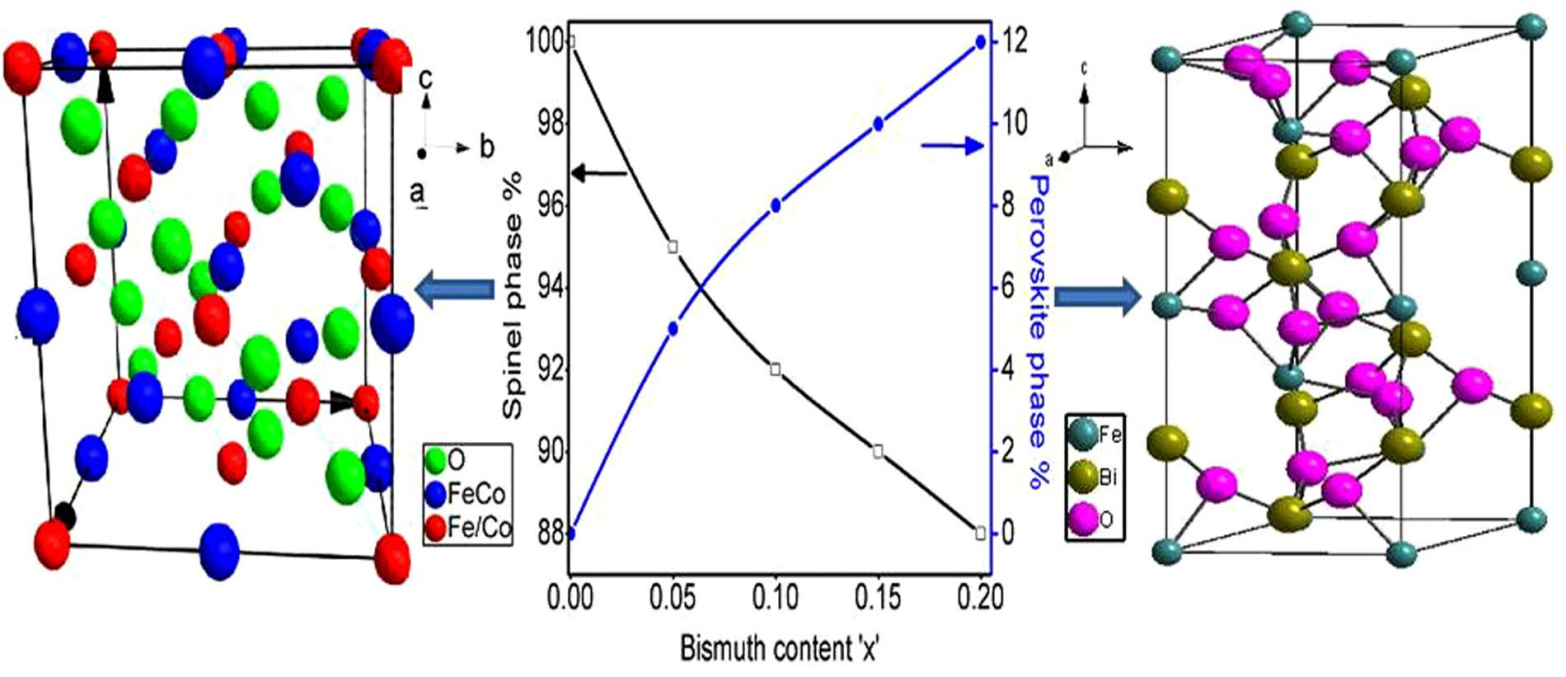
Influence of Bi^3+^-doping on spinel (left) and perovskite (right) phases of CBF.

The crystal structure and phase transition of the samples were confirmed from the XRD patterns. Figure 1 depicts the XRD spectra of CBF ferrite samples obtained for various x values i.e. 0.00, 0.05, 0.10, 0.15 and 0.2. The diffraction patterns and the relative intensity of all diffraction peaks match well to those of JCPDS card number 22–1086, supporting for the formation of CoFe_2_O_4_^30^ phase type. In XRD patterns of x = 0.00 and 0.05 samples, diffraction peaks of other phases are not evidenced, which confirms the formation of single phase cubic spinel structure with space group Fd3m. This suggests that the doping of Bi^3+^ enters the interstices of cubic structure. For x = 0.05, no significant change in the phase of CBF structure has been detected. In accordance with JCPDS card no. 20–0169 with space group-R3c^31^, occurrence of an additional (101) and (110) reflection planes for x ≥ 0.10 Bi^3+^-doping level corroborates the existence of BiFeO_3_. It is to be noted that up to 0.05 doping level of Bi^3+^, the pure cubic spinel phase of CoFe_2_O_4_ is dominating and when Bi^3+^ doping level is ≥0.10, dual i.e. cubic and perovskite phase structures are evolved. The trace amount of Bi^3+^ (1.03 Å) can be embedded into cubic lattice, while the remaining Bi^3+^ could form the perovskite phase. In cubic spinel structure, (220) and (400) planes are sensitive to cation distribution on tetrahedral and octahedral sites, respectively. The cations determine magnetic moment of the ferrite^32, 33^. Inset of Fig. 1 shows an enlarged image of (311) peak where the peak positions are shifted to a higher angle side with substitution of Bi^3+^, suggesting increase of the distortion in the lattice of pure cubic structure^34^. This distortion is may be due to Bi^3+^-substitution which occupies interstices of the ferrite lattice up to x = 0.15. For x = 0.2, the increased amount of Bi^3+^-substitution may create a new phase of perovskite which appears simultaneously with the cubic phase of ferrite. Thus, the trace amount Bi^3+^ may enter into the interstices of the cubic lattice and form perovskite phase simultaneously with the spinel phase. The shifting of (311) peak position by 0.13° supports the formation of CoFe_2_O_4_ in different stoichiometry as a function of the Bi^3+^-substitution. The amount of Bi^3+^, in CoFe_2_O_4_, has been determined by the EDS analysis. The molar ratio of Co^2+^, Bi^3+^ and Fe^3+^ cations are given in Table 1. The Rietveld refinement of structure by using XRD data was processed by using Fullprof suite software. Figure 2 displays the Rietveld refined XRD patterns of CBF where it is clearly evident that, the refined pattern has exhibited two phases; first: the cubic spinel structure with space group Fd3m and the second: the perovskite structure with space group R3c. Figure 3 shows the variation of the spinel and perovskite phases of the CBF as a function of Bi^3+^ **-**doping level. The cubic spinel phase of CBF is decreased and the perovskite phase is increased with increase of Bi^3+^ **-**doping concentration. The unit cells of cubic and perovskite phases are shown on the sides of the graph in Fig. 3. The structure towards left side of the graph shows spinel phase of the cobalt ferrite in which Fe^3+^ is shown to occupy both tetrahedral as well as octahedral sites while Co^2+^ occupies only octahedral sites. The Bi^3+^ cations occupy tetrahedral sites of the perovskite phase (right side of the graph) in which Fe^3+^ cation occupies both tetrahedral and octahedral sites. The phase analysis and structural parameters such as lattice constant obtained from Rietveld refinement of CBF are outlined in Table 1. The variation of lattice constant “*a*” for spinel phase and “*a* = *b*”, “*c*” for perovskite phase is given in Table 1. From the Table 1, it is clear that lattice constants for both the phases have not been changed with increasing Bi^3+^ **-**substitution. In this case, we have replaced Co^2+^ (0.78 Å) by larger Bi^3+^ (1.03 Å). The substitution of larger radii cations generally increases the lattice parameter^31^. However, in this particular case the percentage of substitution of Bi^3+^ is very low. Owing to this, the lattice constant(s), for both the phases remain almost constant. The quality of the refinement was verified by corresponding figure of merit, discrepancy factor (R_wp_), expected values (R_exp_), and goodness fit factor (χ^2^).

**Table 1:** Molar ratio, phase analysis, lattice constant and Rietveld refinement parameter i.e. R_wp_, R_exp_, and χ^2^ of CBF samples.

Comp ‘x’	Molar ratio Co:Bi:Fe	Fd3m Phase (%)	Lattice constant *a* = *b* = **c** (Å)	R3c Phase (%)	Lattice constant, *a* = *b*, *c* (Å)		R_WP_	R_exp_	χ^2^	Deduced formula of the product
0.00	01:00:2	100	8.38	0	—	—	2.34	2.17	1.17	CoFe_2_O_4_
0.05	0.95:0.05:2	95	8.36	5	5.58	13.86	2.41	2.25	1.14	Co_0.95_Bi_0.05_Fe_2_O_4_
0.10	0.9:0.1:2	92	8.37	8	5.57	13.85	2.64	2.37	1.24	Co_0.9_Bi_0.1_Fe_2_O_4_
0.15	0.85:0.15:2	90	8.37	10	5.57	13.82	2.70	2.44	1.23	Co_0.85_Bi_0.15_Fe_2_O_4_
0.20	0.8:0.2:2	88	8.37	12	5.60	13.86	2.87	2.69	1.14	Co_0.8_Bi_0.2_Fe_2_O_4_

### Morphological changes and elemental mapping studies

**Figure 4:**
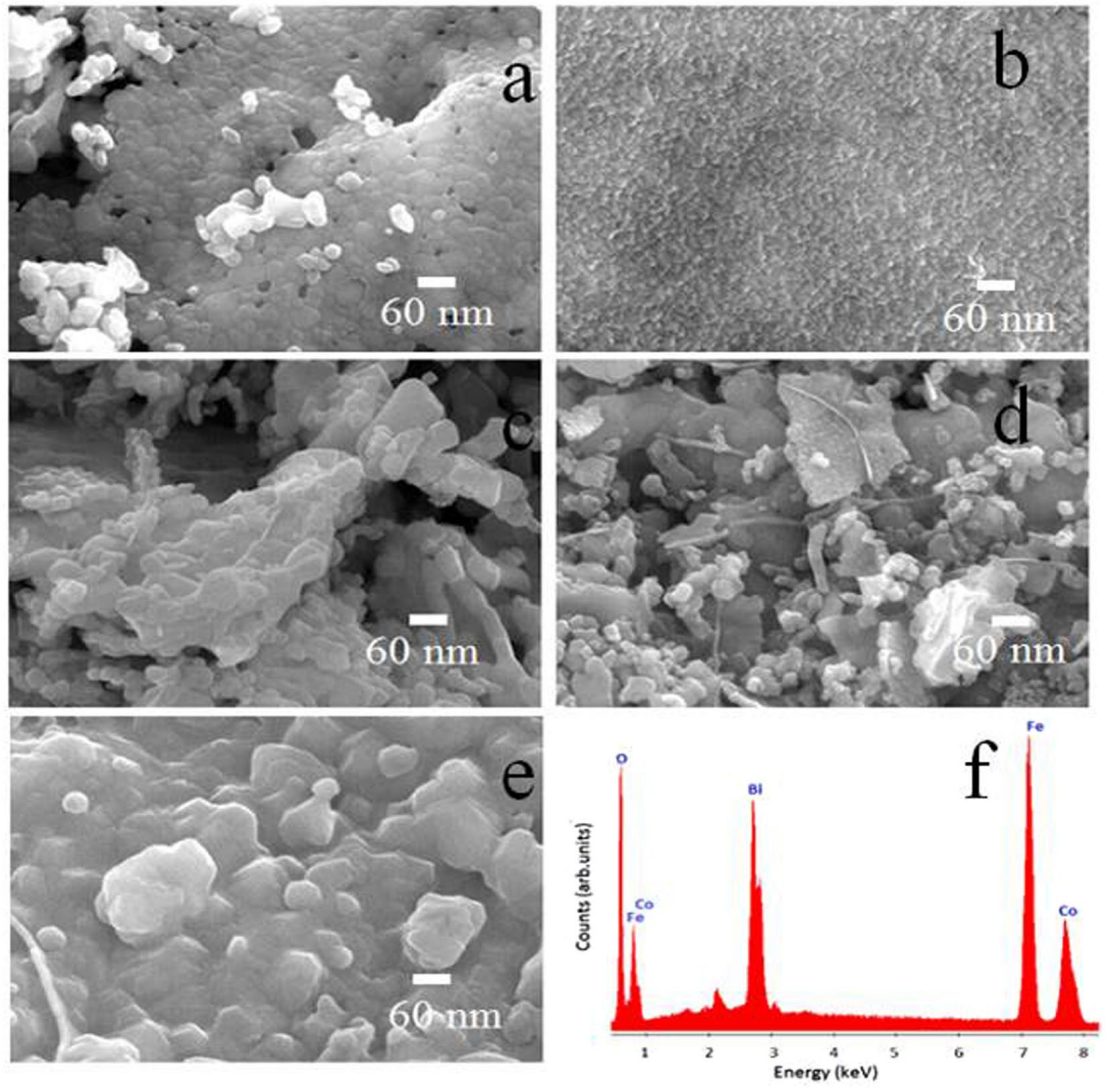
The SEM images of CBF with; (**a**) x = 0.0, (**b**) x = 0.05, (**c**) x = 0.10, (**d**) x = 0.15, (**e**) x = 0.20 and (**g**) EDS (when x = 0.20).

The micro-structural evolution of cobalt ferrite powders at room temperature under various Bi^3+^-doping levels i.e. at x = 0.0, 0.05, 0.1, 0.15, and 0.2 were studied by SEM digital images. Figure 4(a) shows the image of pristine CoFe_2_O_4_ i.e. without Bi^+^-doping. The formation of soft spherical crystallites where majority crystallites are aligned in the same direction by forming a dense structure is vivid from the figure. All crystallites exhibit nearly same size. Figure 4(b) shows image of CBF with 0.05 mol Bi^3+^-doping level where crystallites are smaller in sizes, uniformly distributed and free from the voids or pores. Figure 4(c) shows the image with doping of 0.1 mol of Bi^3+^, where crystallites in the form of plates and rods of bigger sizes are observed. Figure 4(d) and (e) present the surface images of CBF samples for 0.15 and 0.2 mol of Bi^3+^-doping levels, respectively. In previous image, regular crystallites reveal majorly rod-type surface whereas in later case, agglomerated crystallites of different shapes and sizes are evidenced. Figure 4(f) gives EDS spectrum of CBF sample obtained when x = 0.20 where the presence of O, Fe, Co, and Bi as major elements is confirmed, suggesting oxygen and Bi are successfully substituted in the crystal structure. The estimated amounts of (in atomic percentage) O, Fe, Co and Bi in the pure and CBF samples are confirmed from EDS spectra analysis which are tabulated in Table 2. The concentration of Fe has remained nearly same in all powders and the concentration of Co is decreased as the Bi^3+^-doping level is increased. The CBF samples obtained at x = 0.15 and 0.20 exhibit different stoichiometry.

**Table 2:** The stoichiometry (%) of constituent elements present in CBF powders.

Comp. ‘x’	Atomic abundance of elements (%)				
	O	Fe	Co	Bi	Total
0.0	39.93	39.7	29.59	0	100
0.05	51.98	32.14	15.28	0.69	100
0.10	50.32	32.83	15.27	1.57	100
0.15	49.48	33.14	14.81	2.57	100
0.20	47.92	33.08	13.24	5.77	100

### Mossbauer spectra analysis

**Figure 5:**
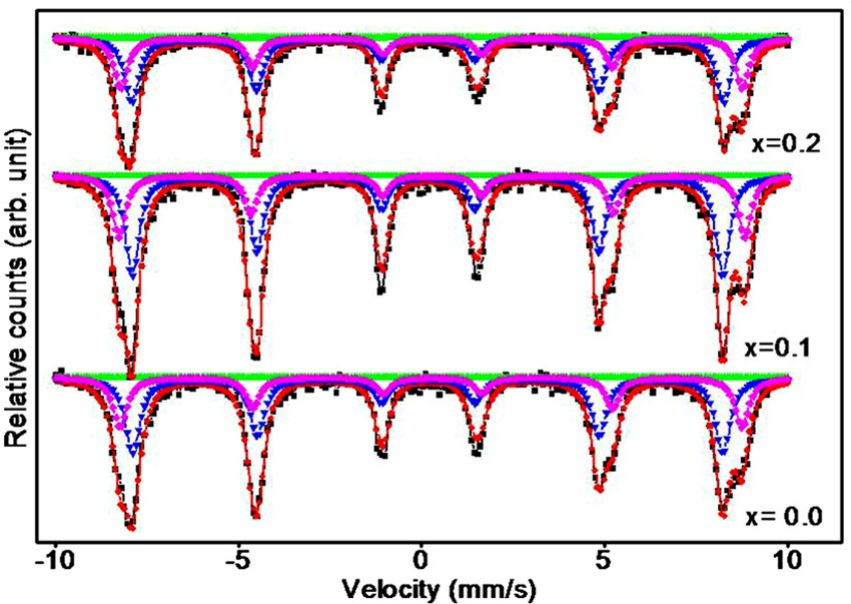
Mossbauer spectra of CBF for x = 0.0, 0.1 and 0.2.

Due to fine energy resolution, Mossbauer spectroscopy can be used to detect even a minute change in the nuclear realm of the iron atoms. In Mossbauer spectroscopy, γ-rays are emitted or absorbed by the crystal without energy loss. So Mossbauer spectroscopy is convenient tool to determine the cation distribution, spin magnetic moment and hyperfine interaction in spinel ferrites^35^. Of all CBF synthesized samples, three (x = 0.0, 0.1 and 0.2) were characterized for Mossbauer analysis at room temperature (Fig. 5). Each spectrum exhibits Zeeman pattern shape with two sub-spectra; one corresponding to Fe ions in tetrahedral A-site and other to Fe ions in octahedral B-site. The hyperfine interactions like isomer shift (IS), quadrupole splitting (QS), magnetic hyperfine field (H_f_), relative area percentage (A), and cation distribution were determined from the analysis of the spectra for three samples and are presented in Table 3. It is observed that IS at A-site is increased and B-site is decreased (by a very small amount) with increasing Bi^3+^ **-**doping concentration which can be explained through the bonding ability of Fe with Co and Bi at both sites. With increasing Bi^3+^ **-**doping level, the occupancy of Fe^3+^ at A-site is decreased while at B-site it is increased^36^. The ions on B-sites are Fe^3+^ and Co^2+^ (0.67 and 0.78 Å) with smaller ionic radius sizes and ions on A-sites are Bi^3+^ and Fe^3+^ (1.03 and 0.67 Å) with larger ionic radius sizes. Due to which there is an expected increase in the orbital’s overlapping of the ions in the A-site and a decrease in the B-sites, resulting in IS changing. The IS value is increased in the A-site because of the replacement of Fe^3+^ with Bi^3+^. The super-transferred hyperfine field components are expected to be strongly influenced by the super-exchange coupling with neighboring ions and the magnetic moments of these ions^37^. In spinel structure, metal ions on octahedral B-site generally have strong super-exchange interactions with six neighboring metal ions on tetrahedral A-sites, while ions on the A-sites can be coupled strongly with twelve neighbouring metal ions on B-sites through the oxygen ion. The B-site ion interacts with only one A-site ion while an A-sites ion interacts with three nearest neighbouring B-sites ions. Because of large separation distance (~3.5 Å), A-sites ions are not expected to have detectable interaction with other A-sites ions (A-A interaction). The B-sites ions may interact with neighboring B-sites ions only by direct overlap. The distance between neighboring atoms lead to weak interactions (B-B interaction). The H_f_ at octahedral B-sites of inverse spinel ferrite is generally 10% greater than that of A-sites and this difference is usually due to covalancy^38^. Table 3 shows the variation of H_f_ at A and B-sites (H_A_ and H_B_) with increasing the Bi^3+^ doping concentration at room temperature. The hyperfine field at both crystallographic sites for samples is decreased when x increased from 0.0 to 0.20 which can be explained with the help of mechanism of supertransferred hyperfine field components. This mechanism is strongly influenced by the super-exchange coupling with neighbouring ions and magnetic moments of these ions^39^. In the present samples of CBF, positions of Fe^3+^ at tetrahedral A-sites are replaced by the Bi^3+^ and super-exchange Bi(A)–Fe(B) between the ions occurs. Huang *et al*.^40^ have reported supertransferred H_f_ mechanism wherein H_f_ value of a Fe^3+^ coupled anti-ferromagnetically with another through superexchange path of 180° has been increased. Thus, it is anticipated that the replacement of A-sites Fe^3+^ with nonmagnetic Bi^3+^ can reduce the hyperfine field at a neighboring B-sites Fe^3+^. Also replacement of B-sites Fe^3+^ and Co^2+^ with nonmagnetic Bi^3+^ can be responsible for hyperfine field to decrease on A-sites. The QS values of CBF are given in Table 3 where the quadrupole splitting for system has shown no variation, indicating Fe^3+^, Co^2+^ and Bi^3+^ symmetry have not been changed between Fe^3+^ and their surrounding with addition of Bi^3+^ in the system. The Mössbauer effect technique was used to investigate these materials. The Mössbauer spectra were used to determine the sites occupancy in the spinel, which are ordered magnetically. The major magnetic interaction is A-B between A-sites and B-sites cations, the A-A and B-B interactions being much weaker. The magnetic field of Fe^3+^ cation depends on the nearest neighbouring cation environment^41^, particularly when it occupies the B-sites. In CoFe_2_O_4_, a broadening of the hyperfine lines from the B-sites due to variations in cation distribution at A-sites is noticed. The relative numbers of Fe^3+^ in A and B-sites were determined from intensity ratios of the outer peaks. From this, the numbers of Co^2+^ and Bi^3+^ on A and B-sites can be determined. The simplest case can be CoFe_2_O_4_ where there is only one Co^2+^ cation with Fe^3+^. From the Mössbauer spectra of CoFe_2_O_4_, the ratio of area of the outer A and B sub-lattice peak is indication of an excess of Fe^3+^ on B-sites and hence fraction of Co^2+^ on A-sites. This distribution can be described by the (Co_0.034_Fe_0.966_) [Co_0.966_Fe_1.03_] O_4_ chemical formula. From Table 3, populations of Fe^3+^ on A-sites and B-sites were estimated. For sample for x = 0.0, 0.1 and 0.2, the number of Fe^3+^ on A and B-sites of CBF are 48.30% and 51.69%, 46.28% and 53.71%, 47.57% and 52.42%, respectively. For pristine sample i.e. for sample with x = 0.0, 3.4% of Co^2+^ occupy tetrahedral A-sites. When Bi^3+^ is substituted, the occupied percentage of Bi^3+^ at A-sites is increased to 7.5% and 4.8% for x = 0.1 and x = 0.2 samples, respectively. With an addition of Bi^3+^ (i.e. x = 0.1), the intensity of outermost A-sites peaks (Fig. 5) is increased by 5.4% with respect to pristine sample (i.e. x = 0.0) and at x = 0.2 intensity of A peak is decreased by 0.73% (with respect to x = 0.0). This trend is consistent with the picture that Bi^3+^ can enter into tetrahedral sites, Co^2+^ enter into the octahedral sites and Fe^3+^ at tetrahedral sites as well as at octahedral sites.

**Table 3:** The IS, QS, H_f_ and A values, obtained from Mossbauer analysis, of CBF for various Bi^3+^ i.e. x values.

Comp ‘x’	Sites	Cation distribution	IS (mm/s)	QS (mm/S)	H_hf_ (kOe)	A (%)
0.0	A	(Co_0.034_Fe_0.966_)	0.27	−0.008	52.1	48.30
	B	[Co_0.966_Fe_1.03_]O_4_	0.29	−0.008	53.3	51.69
0.1	A	(Bi_0.074_Fe_0.925_)	0.27	−0.008	50.1	46.28
	B	[Co_0.9_Bi_0.026_Fe_1.074_]O_4_	0.29	−0.008	54.1	53.71
0.2	A	(Bi_0.048_Fe_0.952_)	0.29	−0.008	50.1	47.57
	B	[Co_0.8_Bi_0.152_Fe_1.048_]O_4_	0.27	−0.008	54.1	52.42

### Magnetic properties

**Figure 6:**
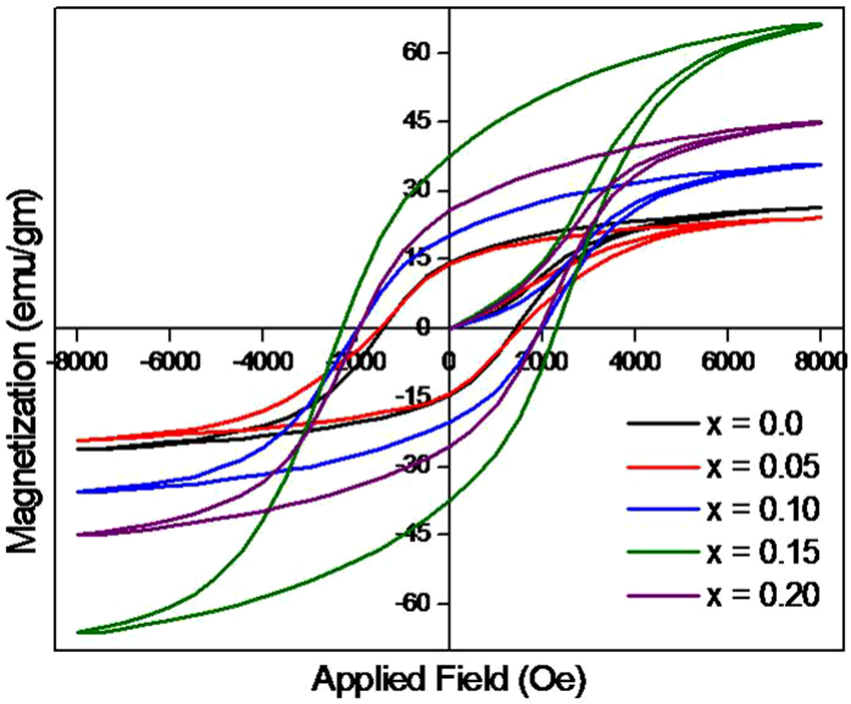
Magnetic hysteresis loops measured for CBF at room temperature for different ‘x’ values.

Magnetic hysteresis loops were recorded at room temperature. Magnetic hysteresis loops of all samples annealed at 500 °C are shown in Fig. 6. The saturation magnetization (M_s_), coercivity (H_c_), remanent magnetization (M_r_) and remanent ratio (R) for all composition of samples are listed in Table 4. It is clear from Fig. 6 that, coercivity and remanent magnetization are increased with Bi^3+^-doping level. The variation of saturation magnetization with Bi^3+^-substitution is shown in Table 4, where increasing (up to x ≤ 0.15) and decreasing (x = 0.2) trends are evidenced. Due to addition of non-magnetic Bi^3+^ ions Fe^3+^ ions from A-sites transferred to B-sites due to which the magnetic moment of A-sites decreases. The net magnetization, being the difference between B and A-sites magnetizations, is increased due to small increase of Fe^3+^ on B-sites. The magnetic moment is supposed to increase with Bi^3+^ content; this could be explained on the basis of magnetic moment of constituent ions. On addition of non-magnetic Bi^3+^, concentration of Fe^3+^ in the A-sites is decreased, as a result magnetic moment of the sites reduces. On B-sites, concentration of Fe^3+^ is increased^42, 43^. Hence on introduction of non-magnetic Bi^3+^ the net magnetic moment up to x = 0.15 is increased. The magnetic moments are dropped for higher values which could be explained on the basis of spin canting. Spin canting is the effect in which non-magnetic substitution on one sub-lattice could lead to a non-collinear or canted spin arrangement on other sub-lattice^44^. As Bi^3+^ (non-magnetic) -content is increased after certain level (x = 0.15), the exchange interactions weaken and the spin magnetic moment of B sub-lattice will no longer be parallel to the spin magnetic moment of A sub-lattice. The decrease in the B sub-lattice moment can be interpreted as a spin departure from co-linearity which causes the effect known as canting^45^. Geller^46^ gave the canting approach in which individual moments on one sub-lattice are canted at different angles. Now out of the two sub-lattices i.e. B and B́, only B́ may have affected by the canting effect. It is presumed that the B́ sub-lattice is formed by the cations of B-sites those are in the neighbourhood of A-sites which contains Bi^3+^. With increasing concentration of Bi^3+^, the canting effect is increased and the spin magnetic moments of B-sites are canted from the direction of net magnetization. The coercivity is increased from 1457 to 2277 Oe with increasing Bi^3+^ which may lead to the fact that H_c_ can be enhanced by enlarging the magnetocrystalline anisotropy. For x = 0.2, number of Co^2+^ is decreased due to increase of Bi^3+^. The Bi^3+^, accommodated at the rhombohedral perovskite lattice sites unable to enter cubic lattice, produces structural distortion in cubic structure, resulting in decrease of coercivity. The remanent ratio, R = M_r_/M_s_ is characteristic parameter of the material. High remanent ratio is desirable for magnetic recording and memory devices^47, 48^. It is an indication of the ease with which the directions of magnetization reorient to nearest easy axis magnetization direction after the magnetic field is removed. Lower value of the remanent ratio is an indication of the isotropic nature of the material. The values of R in the present case are varied from 0.54 to 0.57, showing no significant change in the value with increasing substitution of Bi^3+^.

**Table 4:** The M_s_, H_c_, M_r_ and R values, magnetic hysteresis measurements, of CBF for different ‘x’ values.

Comp ‘x’	M_s_ (emu/g^−1^)	H_c_ (O_e_)	M_r_ (emu/g^−1^)	R
0.0	26.32	1457	14.48	0.54
0.05	27.15	1514	15.21	0.56
0.10	35.60	1993	20.22	0.57
0.15	66.34	2277	37.55	0.57
0.20	44.98	2013	25.53	0.57

## Conclusion

We have demonstrated controlled synthesis of Bi^3+^-doped cobalt ferrite having dual phase (spinel and perovskite) structures, where spinel phase is diminished and perovskite phase is evolved with increase of Bi^3+^-content. The cubic spinel phase is evidenced up to 0.15 Bi^3+^-doping level and for x = 0.2, the perovskite phase is dominating showing impact on structural and magnetic properties of the crystal. The doping of Bi^3+^ has made remarkable and interesting changes in cation distribution, where Bi^3+^ occupy tetrahedral sites thereby replacing Fe^3+^ cations to octahedral sites. This is confirmed from Mossbauer spectra analysis. Saturation magnetization, corecivity and remanence magnetization are increased with increasing doping level of Bi^3+^ and are maximum at x = 0.15. For further increase in doping level to 0.2 of Bi^3+^ discussed magnetic properties are decreased, revealing dominancy of perovskite phase.
